# Dumping Syndrome Post Hiatal Hernia Repair With the LINX Sphincter Augmentation Device

**DOI:** 10.7759/cureus.85893

**Published:** 2025-06-12

**Authors:** Sandon Stokes, Harrison Torgerson, Kenyon Wilson, Mark Twardowski, David Lundy

**Affiliations:** 1 Medical Student, American College of Osteopathic Surgeons Medical Student Section (ACOS-MSS), Rocky Vista University College of Osteopathic Medicine, Ivins, USA; 2 Administrative Department, Western Medical Associates of the Grand Valley, Grand Junction, USA; 3 Internal Medicine, Western Medical Associates of the Grand Valley, Grand Junction, USA; 4 General Surgery, Surgical Associates of the Grand Valley, Grand Junction, USA

**Keywords:** dumping syndrome, gerd, hiatal hernia, linx, surgery

## Abstract

The LINX Reflux Management System is a surgical procedure often done in conjunction with a hiatal hernia repair to manage gastroesophageal reflux disease (GERD). The surgical procedure was developed as an alternative to Nissen fundoplication and is postulated to last indefinitely. Common complications of the LINX device surgery are dysphagia, postoperative pain, and gastric bleeding. One side effect that has not been reported as a complication of the surgery is dumping syndrome. We report on a possible case of dumping syndrome post hiatal hernia repair with LINX magnetic sphincter augmentation diagnosed clinically and confirmed with glucose tolerance tests.

## Introduction

Nissen fundoplication and LINX magnetic sphincter augmentation are both commonly used surgical treatments for the management of hiatal hernia and gastroesophageal reflux disease, but they differ in approach and mechanics. Nissen fundoplication involves wrapping the superior portion of the stomach around the lower esophagus to maintain the lower esophageal sphincter (LES) closure, which helps prevent acid reflux. Nissen fundoplication was first performed in the mid-20th century and is a well-established and effective option for GERD [1,2]. By contrast, LINX involves the implantation of a small, adjustable ring of magnetic beads around the LES to enhance its function without altering the anatomy of the esophagus or stomach. The magnetic force helps prevent reflux by holding the LES closed but allows food and liquid to pass through during swallowing [3]. While Nissen fundoplication may result in more permanent changes to the anatomy, LINX is considered less invasive with a quicker recovery time and potentially fewer long-term complications [4]. 

LINX magnetic sphincter augmentation is associated with a range of side effects. The most common side effect is dysphagia, which occurs in some patients due to the magnetic ring's compression on the esophagus [5]. This can result in a sensation of food sticking in the throat, particularly after eating larger meals. Other reported side effects include pain or discomfort in the chest, regurgitation, and bloating [5]. Although the device is generally well tolerated, some patients may experience increased pressure in the esophagus, leading to a sensation of tightness or even nausea. Another infrequent side effect of LINX is alteration in gastric emptying or the speed at which stomach contents pass into the duodenum. After undergoing LINX implantation patients have demonstrated delayed gastric emptying and rarely increased gastric emptying [6]. Patients reported only the absence of GERD symptom resolution in the cases of increased gastric emptying and symptoms of bloating, lack of GERD symptom resolution, and abdominal pain in the reported cases of delayed gastric emptying [6]. However, there have been no reports of dumping syndrome following LINX surgery. Dumping syndrome is more commonly associated with more invasive gastric and esophageal surgeries such as gastrectomy and esophagectomy [7].

Dumping syndrome is characterized by gastrointestinal, vasomotor, and hypoglycemic symptoms following meals. It is classified into early and late dumping based on predominant symptoms and timing [7]. Early dumping occurs within 30 minutes of eating with predominant symptoms of gastrointestinal distress such as nausea, diarrhea, and abdominal cramping, as well as vasomotor symptoms including palpitations, perspiration, and hypotension. Late dumping occurs one to three hours after eating with predominantly hypoglycemic symptoms such as fatigue, diaphoresis, tremors, and rapid heart rate [7].

Dumping syndrome is an uncommon but recognized complication of Nissen fundoplication [8]. The incidence of dumping syndrome after Nissen fundoplication varies, but it may be more likely in patients who undergo the procedure for more severe reflux or in those who experience an alteration in gastric motility post-surgery [9]. While it is generally not permanent and can be managed with dietary modifications, such as smaller, more frequent meals and reduced sugar intake, some patients may require medical interventions or even revision surgery if symptoms persist [10].

The LINX device procedure for hiatal hernia and GERD does not have any known associations with dumping syndrome. Unlike Nissen fundoplication, which alters the stomach’s anatomy, the LINX device does not change the stomachs’ structure. As a result, patients who undergo the LINX procedure usually retain normal gastric emptying making dumping syndrome unlikely to occur [11,6]. This is one of the postulated advantages of the LINX system, as it avoids some of the complications seen with more invasive surgeries that involve significant anatomical changes [11]. In this case report, we describe an unusual case of dumping syndrome. We highlight its association with recent LINX anti-reflux surgery and describe possible mechanisms for this association based on proposed mechanisms for dumping syndrome following Nissen fundoplication.

## Case presentation

A 73-year-old male presented to general surgery with partially controlled GERD with a moderate-sized paraesophageal hiatal hernia previously diagnosed with esophagogastroduodenoscopy (EGD) and barium swallow study. Robotic laparoscopic surgical intervention was decided on with shared decision-making by the patient. Hiatal hernia repair with the LINX device was undergone in late April with no complications from the surgery. Two week surgical follow-up showed appropriate incisional healing, complete resolution of acid reflux symptoms, and no other complaints.

One month after surgery, the patient presented to the emergency department with symptoms of chest pain, palpitations, diaphoresis, tremors, headaches, fatigue, and dizziness. The chest pain was not relieved by rest or by nitroglycerin. Cardiac workup commenced with serial troponin measurements and repeated electrocardiogram (ECG), both of which were within normal limits. ST-segment elevation myocardial infarction (STEMI) alert was stood down, and additional testing was started. Thyroid-stimulating hormone (TSH) was within normal limits, ruling out thyroid etiology. Complete blood count (CBC) and comprehensive metabolic panel (CMP) showed no abnormalities. Urine metanephrines and fractionated urine catecholamines were within normal limits, ruling out pheochromocytoma. Heart echocardiogram showed no major abnormalities. Lipid panel was within normal limits with the patient being controlled on lipid-lowering drugs since a previous myocardial infarction. The patient had no gallbladder. After discharge, the patient was given a cardiac event monitor that was worn for 28 days and only showed minor premature atrial and ventricular contractions.

**Figure 1:**
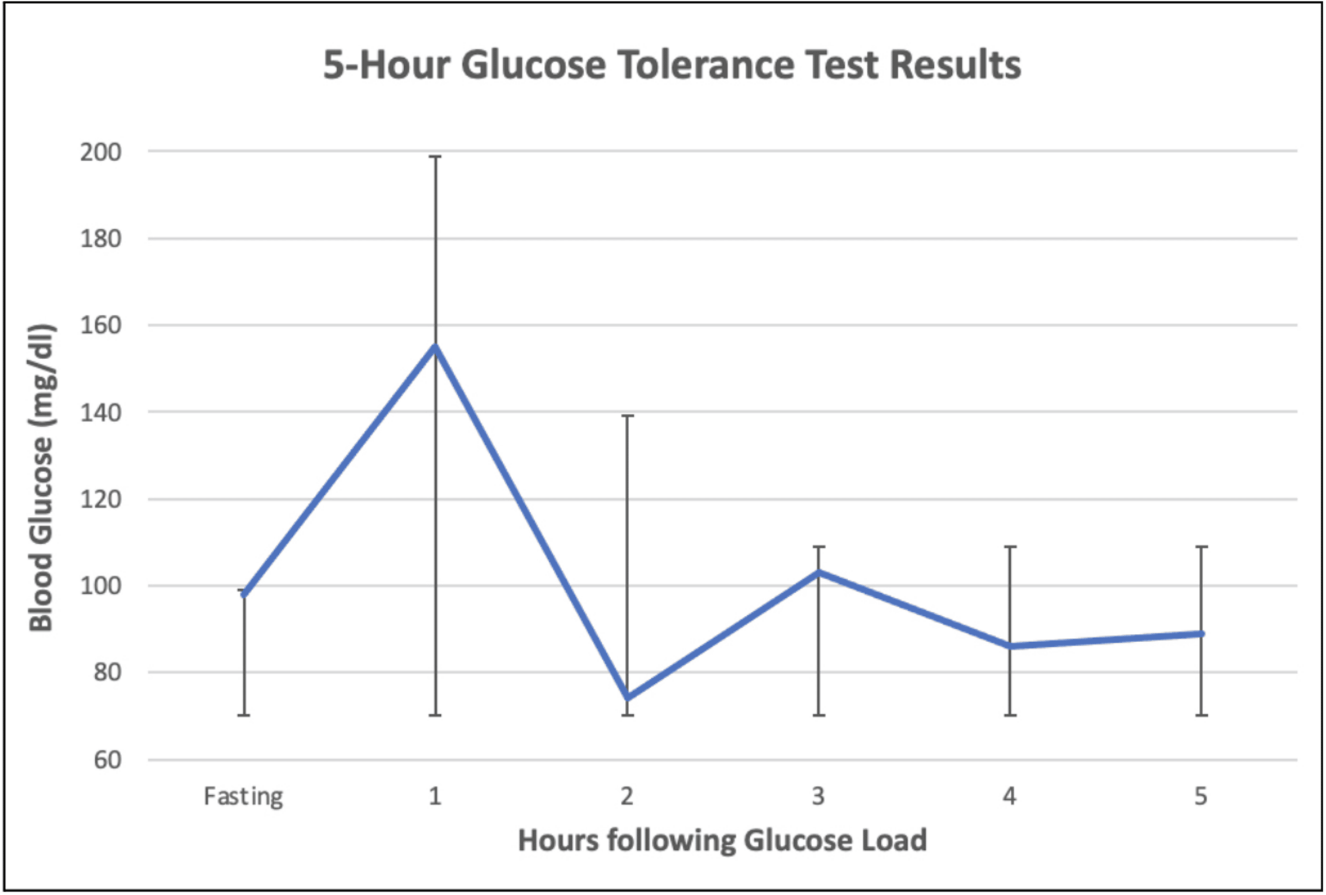
Graph of five-hour glucose tolerance test results showing a rapid decrease in blood glucose levels between the one- and two-hour marks. Reproduction of patient symptoms seen around the two- to three-hour mark.

The patient was seen by primary care a couple of weeks after the emergency department visit. He now reports episodes of chest pain, palpitations, diaphoresis, and dizziness that happen almost everyday about two hours after lunch. Hypoglycemia was suspected, and the patient underwent a two-hour and then a five-hour glucose tolerance test using a 75 gm glucose load, with results shown in Table 1 and Figure 1. The threshold for normal glycemic values in Table 1 shows that the threshold for hypoglycemia was 70 mg/dl with the upper thresholds changing dynamically during the test. From hour 1 to hour 2, there was a steep drop in blood sugar, to albeit non-hypoglycemic levels, with reproduction of previous concerning symptoms around hours 2 and 3, indicating a drop in blood sugar as a part of the likely etiology. The patient was seen in the clinic again and advised to make diet changes necessary to keep blood sugars stable throughout the day.

**Table 1:** Five-hour glucose tolerance test results showing a rapid decrease in blood glucose levels between the one- and two-hour marks. Reproduction of patient symptoms seen around the two- to three-hour mark.

Test	Result (mg/dL)	Reference (mg/dL)
Glucose, fasting	98	70-99
Glucose, hour 1	155	70-199
Glucose, hour 2	74	70-139
Glucose, hour 3	103	70-109
Glucose, hour 4	86	70-109
Glucose, hour 5	89	70-109

Months later, in October, the patient was seen in the clinic reporting five to six weeks of diarrhea within 30 minutes after meals and mild symptoms of palpitations, diaphoresis, and dizziness. At this time, the patient underwent testing of stool cultures,* Helicobacter pylori * screening, CBC, CMP, and colonoscopy to rule out microscopic colitis and other gastrointestinal causes. All tests were negative or within normal limits. These symptoms of diarrhea immediately after meals gradually subsided, and the patient reverted back to having chest pain, palpitations, diaphoresis, and dizziness two to three hours after meals.

To make the case more complicated, the patient was hospitalized in early November for a cecal volvulus and treated surgically with a right hemicolectomy and ileocolic anastomosis. The patient’s vasomotor and gastrointestinal symptoms continued throughout the healing process and afterward.

After adequate healing time, the patient was seen back by the surgeon in late November. The patient scored 10 on the Sigstad scoring system with a score >7 suggesting dumping syndrome and a score <4 suggesting a consideration for other diagnoses [12]. The Sigstad scoring system is shown in Table 2. With the extensive workup done and the patient’s Sigstad score, the most likely diagnosis was postulated to be a late-phase dumping syndrome, followed by some early-phase dumping syndrome symptoms that then reverted back to a late-phase dumping syndrome.

**Table 2:** Sigstad scoring system. A score >7 suggests a diagnosis of dumping syndrome; a score <4 suggests an alternate diagnosis. Reference: [12]

Sigstad scoring system	
Postprandial symptom	Score
Shock	+5
Syncope, unconsciousness	+4
Desire to lie or sit down	+4
Breathlessness, dyspnea	+3
Weakness, exhaustion	+3
Sleepiness, drowsiness, apathy, falling asleep	+3
Palpitations	+3
Restlessness	+2
Dizziness	+2
Headaches	+1
Warmth, clammy skin, sweating, or pallor	+1
Nausea	+1
Abdominal fullness	+1
Borborygmus	+1
Eructation	-1
Vomiting	-4

The patient was advised to eat less simple carbohydrates, five to six smaller meals throughout the day, and wait to drink fluids 30 minutes after meals. Finally, the patient underwent a mixed meal scintigraphic study to assess gastric emptying and evaluate suitability for medical management, which revealed delayed gastric emptying, as shown in Table 3, with no symptoms of gastroparesis. The patient’s vasomotor symptoms continued, but did improve significantly with dumping syndrome diet modifications.

**Table 3:** Gastric scintigraphic study showing delayed gastric emptying by percent retention above maximal normal limits from hours 2-4.

Time	Percent retention	Normal limits
Start of test	100%	Lower: 70%
Hour 1	88%	Lower: 30%; upper: 90%
Hour 2	73%	Upper: 60%
Hour 3 1/2	31%	Upper: 30%
Hour 4	21%	Upper: 10%

Dumping syndrome dietary modifications have benefited the patient greatly after much trial and error. A more strict adherence to dumping syndrome diet and patient education has now led to a near-complete resolution of symptoms. Other medical management options such as administration of octreotide or acarbose were considered to be the next-line therapy should diet modification fail. If these medical management options do not produce effective treatment, surgical management could be a last line treatment option if symptoms continue to be unmanageable.

## Discussion

The current diagnosis of dumping syndrome is primarily clinical and established with symptom-based questionnaires, such as the Sigstad scale [12]. Laboratory confirmation of the diagnosis has classically involved either gastric emptying scintigraphy or oral glucose tolerance testing. Rapid gastric emptying is a key component of the mechanism of dumping syndrome; however, gastric emptying scintigraphy is no longer used for diagnosis [12,13]. Recent research has shown low sensitivity and specificity for gastric emptying studies in diagnosing dumping syndrome and has suggested that initial rapid emptying could lead to overall delayed emptying on scintigraphy [12,13]. Due to this, the preferred diagnostic test for dumping syndrome is the modified glucose tolerance test, measuring blood glucose concentration, hematocrit, pulse rate, and blood pressure at 30-minute intervals for three hours [12]. The cut-off for hypoglycemia in most recent research suggests 50 mg/dl for the diagnosis of late dumping syndrome, while some studies have used 60 mg/dl and 70 mg/dl [12]. 

With these diagnostic criteria in mind, this case represented a complex diagnosis. The patient scored 10 on the Sigstad scale, suggesting dumping syndrome as the likely diagnosis. The confirmatory tests were not as straightforward. The gastric emptying scintigraphy demonstrated delayed gastric emptying. Although this test is no longer used due to low sensitivity and specificity, it is not the typical expected result [10,12,13]. An important differential with this result is gastroparesis. However, the patient exhibited no symptoms of gastroparesis such as bloating, emesis, or nausea following meals [6]. 

Other considerations for the scintigraphic test result include timing and unrelated gastrointestinal surgery. The study was initiated at 7 AM, while the majority of the patient symptoms typically develop later in the afternoon, two to three hours after lunch. The patient’s gastric motility may be slower in the morning while fasting, when compared to the afternoon motility levels. It has been found that gastric motility can be influenced by a number of factors, including parasympathetic activation through the vagus nerve, various hormone levels, and higher central brain centers [14,15]. In addition to these details, the patient had a cecal volvulus in early November, treated with right hemicolectomy and ileocolic anastomosis that has markedly changed the patient’s anatomy and caused significant inflammation. The inherent variability in gastric emptying studies, along with these factors, accounts for delayed emptying in this case rather than the expected rapid emptying. 

The oral glucose tolerance test largely supports the diagnosis of dumping syndrome, but also has some limitations. The test reproduced the patient's symptoms two hours following the glucose load. At this time, there was a large decrease in the patient’s blood sugar from 155 mg/dl at hour 1 to 74 mg/dl at hour 2, with a reproduction of the patient’s symptoms. The major limitation to this test is that the lower level of 74 mg/dl does not meet the threshold for hypoglycemia. The major differences between this glucose tolerance test and the modified glucose tolerance test include the following: this test measured hourly glucose levels rather than every 30 minutes; hematocrit, blood pressure, and pulse rate were also not monitored throughout the test. 

All of this information together leads us to the diagnosis of dumping syndrome for this patient. They had symptoms consistent with dumping syndrome, evidenced by their Sigstad score. The patient experienced a reproduction of their symptoms during the glucose tolerance test at their lowest recorded glucose level. Perhaps, most importantly, their symptoms have largely resolved following dumping syndrome-specific dietary changes.

Dumping syndrome is a rare complication of the Nissen fundoplication surgery for GERD, as demonstrated in a number of cases [8,16,17]. It has been postulated that this is due to the reduced stomach fundus size in Nissen fundoplication, leading to an increased emptying rate of the stomach. An additional hypothesis is that damage to the vagus nerve can cause decreased fundal accommodation and, in turn, cause increased gastric motility and possibly dumping syndrome [8]. The LINX procedure does not decrease the size of the stomach and mainly provides gentle pressure on the lower esophagus, making reduced fundal size an unlikely cause of dumping syndrome. Although the vagus nerve is isolated and preserved during LINX placement, there is a possibility that its inadvertent damage could contribute to the development of dumping syndrome [3]. Mechanisms such as inflammation, scar tissue entrapment, and even partial transection may all represent possible mechanisms of injury to the vagus nerve and its branches, leading to dumping syndrome [18]. However, it is important to note that the cause of dumping syndrome in this case is unknown, and there is merely an association between this patient’s LINX magnetic sphincter augmentation and the development of dumping syndrome.

## Conclusions

This case represents a complex clinical and laboratory case of dumping syndrome following hiatal hernia repair with LINX device implantation. The patient's symptoms, glucose tolerance test results, and extensive diagnostic workup collectively support the diagnosis of dumping syndrome. Although dumping syndrome is an unreported association following LINX implantation, it is essential for surgeons performing hiatal hernia repair with LINX, as well as primary care providers conducting postoperative visits, to be aware of this potential association. Increased awareness will lead to earlier diagnosis, more effective treatment, and ultimately improved patient outcomes.
